# The 'genetic zipper' method offers a cost-effective solution for aphid control

**DOI:** 10.3389/finsc.2024.1467221

**Published:** 2024-12-11

**Authors:** Vol V. Oberemok, Yelizaveta V. Puzanova, Nikita V. Gal’chinsky

**Affiliations:** ^1^ Department of General Biology and Genetics, Institute of Biochemical Technologies, Ecology and Pharmacy, V.I. Vernadsky Crimean Federal University, Simferopol, Republic of Crimea; ^2^ Laboratory of Entomology and Phytopathology, Dendrology and Landscape Architecture, Nikita Botanical Gardens—National Scientific Centre of the Russian Academy of Sciences, Yalta, Republic of Crimea

**Keywords:** ‘genetic zipper’ method, oligonucleotide insecticides, contact unmodified antisense DNA (CUAD) biotechnology, cost-effective aphid control, DNA-programmable plant protection

## Abstract

Twenty years ago, it was difficult to imagine the use of nucleic acids in plant protection as insecticides, but today it is a reality. New technologies often work inefficiently and are very expensive; however, qualitative changes occur during their development, making them more accessible and work effectively. Invented in 2008, contact oligonucleotide insecticides (olinscides, or DNA insecticides) based on the CUAD (contact unmodified antisense DNA) platform have been substantially improved and rethought. The main paradigm shift was demonstrating that unmodified antisense DNA can act as a contact insecticide. Key breakthroughs included identifying convenient target genes (rRNA genes), mechanism of action (DNA containment), and discovering insect pests (sternorrhynchans) with high susceptibility to olinscides. Today, the CUAD platform possesses impressive characteristics: low carbon footprint, high safety for non-target organisms, rapid biodegradability, and avoidance of target-site resistance. This next-generation class of insecticides creates opportunities for developing products tailored for specific insect pest populations. The ‘genetic zipper’ method, based on CUAD biotechnology, integrates molecular genetics, bioinformatics, and *in vitro* nucleic acid synthesis. It serves as a simple and flexible tool for DNA-programmable plant protection using unmodified antisense oligonucleotides targeting pest rRNAs. Aphids, key pests of important agricultural crops, can be effectively controlled by oligonucleotide insecticides at an affordable price, ensuring efficient control with minimal environmental risks. In this article, a low-dose concentration (0.1 ng/µL; 20 mg per hectare in 200 L of water) of the 11 nt long oligonucleotide insecticide Schip-11 shows effectiveness against the aphid *Schizolachnus pineti*, causing mortality rate of 76.06 ± 7.68 on the 12^th^ day (p<0.05). At a consumption rate of 200 L per hectare, the cost of the required oligonucleotide insecticide is about 0.5 USD/ha using liquid-phase DNA synthesis making them competitive in the market and very affordable for lab investigations. We also show that non-canonical base pairing G_olinscide_: U_rRNA_ is well tolerated in aphids. Thus, non-canonical base-pairing should be considered not to harm non-target organisms and can be easily solved during the design of oligonucleotide insecticides. The ‘genetic zipper’ method, based on CUAD biotechnology, helps quickly create a plethora of efficient oligonucleotide pesticides against aphids and other pests. Already today, according to our estimations, the ‘genetic zipper’ is potentially capable of effectively controlling 10-15% of all insect pests using a simple and flexible algorithm.

## 1 Introduction

Unmodified DNA, as a programmable molecule and polymer of natural origin, has always attracted researchers. Unfortunately, for a long time it was believed that unmodified oligonucleotides are toxic to cells and degraded quickly in all eukaryotic cells under the action of nucleases (1), including insects (2). Some articles literally stated that unmodified (phosphodiester) antisense oligonucleotides should not be used for these experiments (3). The mode of action of unmodified antisense DNA and its potential application as contact insecticide have not been investigated on insect pests, and no attempts were made to test this hypothesis until the beginning of the 21st century. An unexpected and surprising turn with unmodified antisense oligonucleotides in plant protection came in 2008, when it was shown that short unmodified antisense DNA has significant insecticidal effect on insect pests (4). For the first time, the equivalence between antisense oligodeoxyribonucleotides and contact insecticides was established in experiments with spongy moth *Lymantria dispar*, which led to the development of the CUAD (contact unmodified antisense DNA) platform (5–8). The first 18–20 nt long oligonucleotide insecticides, based on anti-apoptotic genes, showed effectiveness on virus-free and nuclear polyhedrosis virus-infected spongy moth caterpillars (9). This discovery opened up an entirely new dimension in insect pest control using nucleic acids as contact insecticides. Scientists studying RNAi also picked up this idea three years later when Wang et al. (10) successfully applied double-stranded RNA fragments as contact insecticides in insect pest control for the first time (10).

In 2019, this innovation was substantially improved and rethought (11). The CUAD biotechnology has a number of features that distinguish it from all modern classes of chemical insecticides and plant protection technologies developing today: unmodified antisense DNA as active substance, DNA containment as mechanism of action, insect pre-rRNA and rRNA as target (12–14). Oligonucleotide insecticides are based on ‘genetic zipper’ method acting through formation of complementary DNA olinscide-rRNA duplex (resembles zipper mechanism) that ‘zips’ target rRNA expression and leads to death of pests. Oligonucleotide insecticides (briefly, olinscides, or DNA insecticides) act on sternorrhynchans through DNA containment mechanism consisting of 2 steps: 1) rRNA and/or pre-rRNA ‘arrest’ and hypercompensation of target rRNA; 2) target rRNA and/or pre-rRNA degradation recruiting RNase H (13–15). Use of insect pest pre-rRNA and mature rRNA as target leads to high efficiency of oligonucleotide insecticides, since pre-rRNA and mature rRNA comprise 80% of all RNA in the cell. The degradation of ribosomal RNA inevitably leads to disruption of protein biosynthesis and the death of insect pests (9). If insecticide resistance occurs, different strategies can be applied. Generally, new and efficient olinscides can be easily re-created displacing target site to the left or to the right from the olinscide-resistant site of target mature rRNA and/or pre-rRNA (11, 13). According to our investigations, contact delivery of unmodified antisense DNA (CUAD) is much more efficient (16) than oral delivery of unmodified antisense DNA (ODUAD) because of active DNases present in the digestive tract of insects (17, 18).

While 1^st^ step of discovered DNA containment mechanism (rRNA ‘arrest’ accompanied with rRNA hypercompensation) was completely unknown before, 2^nd^ step (degradation of rRNA) recruiting RNase H was partially known but not for rRNAs (19, 20). Historically, some principles of the practical application of antisense oligonucleotides (ASOs) for chemical reactions on biopolymers were first formulated in Novosibirsk (Russia) by N. Grineva in 1967. In the case of nucleic acids, it was promising to obtain compounds, containing a reactive group bound to oligonucleotide residue capable of specific base pairing with the complementary nucleotide sequence (21). In 1977, using this antisense approach to modify valine tRNA, N. Grineva and colleagues demonstrated that the method allows alkylation with a reagent bound to the corresponding oligonucleotide at certain points along the valine tRNA (22). These first steps were quite different from the research work currently being carried out using antisense oligonucleotides in agriculture and medicine, but they laid the groundwork for the possibility of regulating gene expression in living organisms using antisense oligonucleotides. Later, in 1978, P. Zamecnik and M. Stephenson showed that modified antisense DNA hinders reproduction of Rous sarcoma virus in chicken embryo fibroblasts in a sequence-specific manner (23). In 1979, H. Donis-Keller presented results showing that RNase H cleaves the RNA strand in RNA–DNA heteroduplexes in a site-specific manner (24). The development of antisense technologies has long been primarily focused on medicine using modified antisense oligonucleotides (25). After 20 years of research with modified antisense oligonucleotides, in 1998, the FDA licensed the first drug, Vitravene (Fomivirsen), based on the 21-mer phosphorothioate oligonucleotide (26). This area of medicine continues to progress and has already seen the registration of other important drugs (27). In any case, it had not been shown until 2008 that DNA can function as insecticide and there were no attempts to investigate precise mechanisms of action of unmodified antisense DNA on insect cells, including rRNA as a target.

To date, contact unmodified antisense DNA (CUAD) biotechnology is the only platform that successfully uses short unmodified antisense DNA for plant protection (11, 13, 14). The CUAD platform works best on hemipterans from suborder Sternorrhyncha (11, 12). The main paradigm shift with unmodified antisense DNA was in showing that it can be a contact insecticide. The main breakthroughs in the development of this approach were in finding the most convenient target genes (rRNA genes), showing mechanism of action (DNA containment) and in searching up insect pests (sternorrhynchans) with high susceptibility to this approach. n the last few years, CUAD biotechnology based on oligonucleotide pesticides has been established as a powerful “genetic zipper” method against soft scale insects, armored scale insects, psyllids, mealybugs, aphids, and mites, opening new frontiers in DNA-programmable plant protection based on contact application of deoxyribonucleic acid (12, 15). Obtained data suggest that short antisense DNA sequences via DNA containment (DNAc) mechanism can participate in regulation of rRNA expression by complementary interaction with cell DNA (direct rDNA master regulation of rRNA expression) and viral DNA (direct rDNA master regulation of rRNA expression by viral DNA, or rRNA switchboard mechanism) (14). Also DNAc can be recruited in innate immunity system (13) against ssDNA viruses for which hemipteran insects serve as major vectors (28, 29) and also against DNA viruses that normally infect them (30).

Because of very efficient and easy algorithm, DNA-guided ‘genetic zipper’ method (CUAD biotechnology) is a unique and very potent alternative to other antisense approaches in insect pest control based on duplexes of unmodified nucleic acids and RNA-guided nucleases: RNA interference and CRISPR/Cas9. Innovative insect pest control technologies (RNAi, CUAD, CRISPR/Cas9) are based on formation of duplexes of unmodified nucleic acids (RNAi: guide RNA-mRNA; CUAD: guide DNA-rRNA; CRISPR/Cas9: guide RNA-genomic DNA) and action of nucleic acid-guided nucleases (RNAi: Argonaute, briefly Ago; CUAD: RNase H; CRISPR/Cas9: CRISPR associated protein 9, briefly Cas9) (31). While RNAi and CRISPR/Cas9 were not discovered on insect pests and initially had fundamental importance rather than practical one, CUAD biotechnology was discovered on insect pests as practical tool and recently fundamental importance of DNA containment mechanism played in rRNA biogenesis was revealed (9, 13, 14, 32). To date, while RNAi and CRISPR/Cas9 are excellent tools for manipulations with unmodified nucleic acids in laboratory, they do not have easy algorithms for creation of end-products for insect pest control; each separate case is special and usually is sorted out using trial and error method.

Oligonucleotide insecticides possess low carbon footprint, high safety for non-target organisms, rapid biodegradability in ecosystems, and avoidance of target-site resistance. While current chemical insecticides require days, months and even years for biodegradation by bacteria and fungi, oligonucleotide insecticides are substantially biodegraded within hours in the presence of nucleases (33). Olinscides have the potential to complement the existing insecticide market and set an eco-precedent for crop protection products where the effectiveness of the insecticide will be determined by its safety for non-target organisms (33). The advantage of using natural oligomers, unmodified antisense oligonucleotides, seems to be the safest way, since the cells of all living organisms contain ubiquitous nucleases that can neutralize them (8). Consequently, for oligonucleotide insecticides there is no need to look for methods of accelerated biodegradation. The principle of using oligonucleotide pesticides is that they must have enough time to act in the right place and on the right organism before their rapid biodegradation (and they successfully do this on sternorrhynchans and other pests). In contrast, due to resistance to biodegradation conventional chemical insecticides have too much time for their action not only in the right place and not only on the right organism (33). Consequently, majority of chemical insecticides were banned in one way or another after decades or years of use in plant protection, when effective competitors with proven or supposed greater safety appeared (34). Recently, the idea of oligonucleotide insecticides attracted the attention of scientists and experts in plant protection (35–40) and to a certain extent the affordability of such insecticides remained in question. If in the near future a balance is found between the effectiveness and cost of such pest control agents, then the insecticide market will be replenished in abundance with species-, genus-, and family-specific oligonucleotide insecticides.

Our research team decided to lower the price of olinscides and find a group of serious insect pests on which oligonucleotide insecticides at low concentrations could have a significant insecticidal effect. Aphids from subfamily Lachninae turned out very susceptible to low-dose concentrations of oligonucleotide insecticides. Generally, aphids (Hemiptera: Aphididae) are significant economic pests that are found globally. Aphids feed on phloem (41) and cause substantial economic losses mainly spreading plant viruses, and producing honeydew (42–44). Among aphids around 100 species are considered to be agricultural pests of a wide range of crops. They are major insect pests of various plants, including alfalfa, wheat, potato, sugar beet and tobacco (45). The damage caused by aphids amounts to hundreds of millions of dollars a year (46). Their management is challenging because the mobility of aphids is extremely high (47). Also these pests reproduce predominantly asexually (48), one female leaves 10-90 offspring in 7-10 days and therefore, theoretically, could produce billions of offspring in one growing season in the absence of mortality factors (49). Chemical insecticides have been used to control aphids, and they quickly develop resistance to various classes of chemical insecticides, including neonicotinoids, carbamates, organophosphates, organochlorines, and pyrethroids (50). This prompts the search for new insecticides with advanced characteristics and multi-decade utility.

In this article we use aphid *Schizolachnus pineti* for the experiments. *S. pineti* is a serious pest of *Pinus* spp., but especially on young Scots pine (*Pinus sylvestris*) where it forms dense colonies in rows along the previous year’s needles (51). The goal of this article is to show insecticidal effect on *S. pineti* using low concentrations of olinscides and provide cost-effective solution for aphid control based on the DNA-programmable ‘genetic zipper’ method.

## 2 Materials and methods

### 2.1 Origin of material

**Figure 1:**
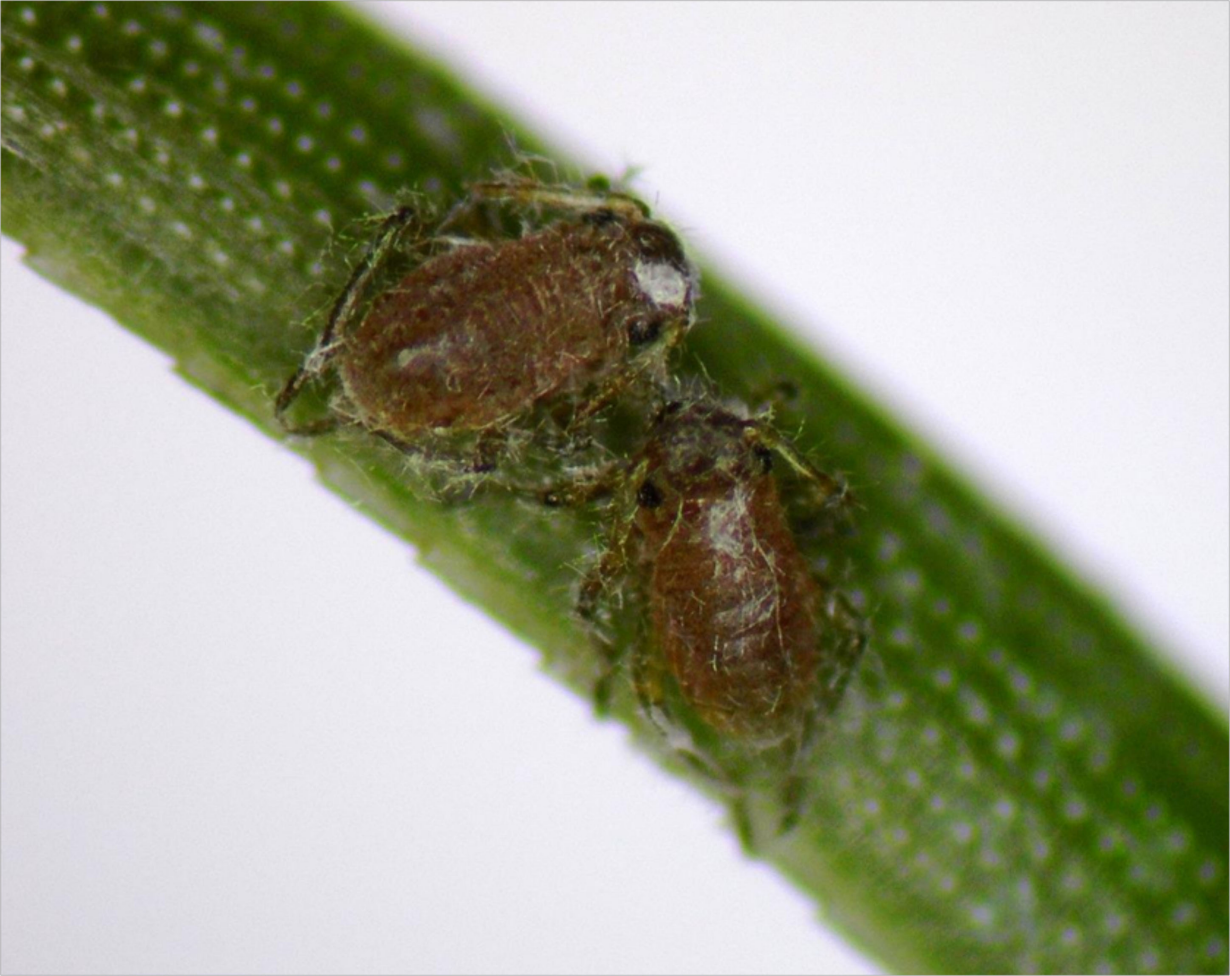
*S. pineti* on needle of *P. sylvestris*.

Individuals of *S. pineti* were collected from pine forest in Crimea. The experiments with *S. pineti* (**Figure 1**) were carried out in laboratory conditions at room temperature (25°C) and 50% humidity on shoots of *Pinus sylvestris* L. (Coníferae: Pinaceae). Shoots of trees with *S. pineti* were immersed in water and randomized; 3 shoots were taken for each experimental group; there were 100-120 aphids on each shoot in all experimental groups including water-treated Control (300-360 insect individuals per 3 shoots of *P. sylvestris* in each replicate for each group of the experiment). Experiments were carried out in triplicate.

### 2.2 Design, synthesis, and application of oligonucleotide insecticide Schip-11

**Figure 2:**
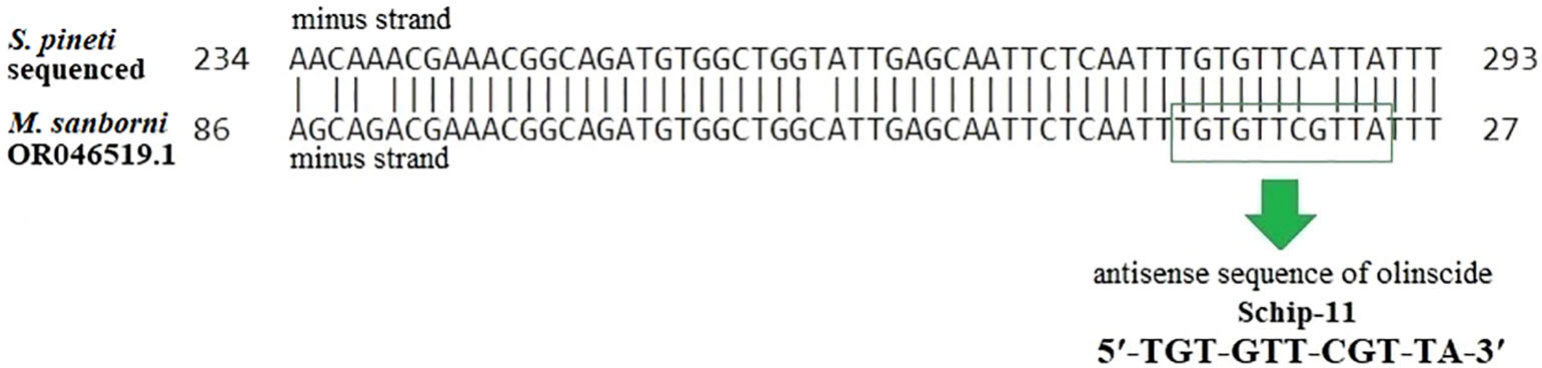
Alignment of the sequenced DNA fragment of *S. pineti* collected from nature and fragment of ITS2 region of rDNA of *M. sanborni* (GenBank: OR046519.1) performed using ClustalW 2.0.3.

We designed oligonucleotide insecticide Schip-11 5′-TGT-GTT-CGT-TA-3′ which is almost complementary (at 4^th^ position of olinscide, G instead of A) to the ITS2 region of pre-rRNA of *Schizolachnus pineti* (**Figure 2**). We decided to use Schip-11 sequence to find out if non-canonical base pairing G_olinscide_: U_rRNA_ in aphids is well tolerated as seen in scale insects (13). Also G:U base pairs are among the first-identified and most frequently occurring non-canonical Watson-Crick interactions in structured RNAs (52), thus, non-canonical base-pairing should be taken into consideration during creation of olinscides.

The sequence of olinscide was synthesized using ASM 800E DNA synthesizer (BIOSSET, Novosibirsk, Russia) according to standard phosphoramidite synthesis procedure. The synthesis was carried out in the direction from the 3′ to the 5′ end. After completion of all cycles of synthesis, the target oligonucleotide was removed from the solid-phase support; the removal of the protective groups was carried out overnight at 55°C in a concentrated ammonia solution (analytical grade, “Vekton”, Saint Petersburg, Russia). Purification of the synthesized olinscide Schip-11 was performed on OPS-12 cartridges used for purification of oligonucleotides (BIOSSET, Novosibirsk, Russia). A BactoSCREEN analyzer based on a MALDI-TOF mass spectrometer was used to determine the quality of produced olinscide Schip-11 (Litech, Moscow, Russia). The ratio of mass-to-charge (*m*/*z*) of olinscide Schip-11 was measured as positive ions with 3-hydroxypicolinic acid as a matrix on a LaserToFLT2 Plus device (UK) in a ratio of 2:1. The theoretical *m*/*z* ratio was calculated using ChemDraw 18.0 software (ChemDraw, Cambridge Soft, USA) and differed by no >10 units with the resulting *m*/*z* ratio. Dilution in nuclease-free water to a required concentration was carried out on NanoDrop Lite spectrophotometer (Thermo Fisher Scientific, Waltham, MA, USA).

Concentrations of 200 ng/μL and 0.1 ng/μL of olinscide Schip-11 in nuclease-free water was applied on *P. sylvestris* leaves using hand sprayer (10 ml of water solution per m^2^ of leaves). As a control, water-treated group was used. Mortality was recorded every day during 4 days for insects treated with Schip-11 in concentration 200 ng/µL and during 12 days for insects treated with Schip-11 in concentration 0.1 ng/µL. Effectiveness of olinscide Schip-11 was calculated by dividing the number of dead insect individuals by the total number of insect individuals on the shoot and multiplying by 100%, winged insect individuals were excluded from calculations.

### 2.3 Target gene expression

Isolation of total RNA was carried out according to manufacturer’s instructions using ExtractRNA reagent (Evrogen, Moscow, Russia). RNA extraction was carried out in three replicates. A MMLV RT kit was used to perform first-strand cDNA synthesis (Syntol, Moscow, Russia), following the manufacturer’s instructions. For cDNA synthesis, 10 μl of RNA was taken at a concentration of 20 ng/µl. PCR reactions were carried out on 5 μL of cDNA using 10 μL of SYBR Green Master Reaction Mix reagent (Syntol, Moscow, Russia), 7 μL of ddH_2_O (Syntol, Moscow, Russia), 1 μL of MgCl_2_ and 1 μL (80 ng/μL) of each specific primers SP_F 5′-ACG-ACA-ACA-TGC-GTG-TAC-C-3′ and SP_R 5′-GTC-CCA-CAG-TCC-GCT-TCTC-3′. The following PCR program was applied: 10 min of initial denaturation at 95°C, followed by 30 cycles with 10 s of denaturation at 95°C, 15 s of annealing at 62°C, and 20 s of elongation at 72°C was used for amplification on a LightCycler^®^ 96 Real-Time PCR System (Roche, Basel, Switzerland). The expression of the target gene was evaluated on the 1^st^ and 3^rd^ day after treatment with Schip-11.

### 2.4 DNA sequencing

Primers, forward 5′-CGT-CGT-AAC-CTT-GCC-CTC-TT-3′ and reverse 5′-CGG-GGA-CAT-CGT-GAT-TTT-GG-3′, were used for PCR. PCR reactions were carried out on 5 μL of cDNA using 10 μL of SYBR Green Master Reaction Mix reagent (Syntol, Moscow, Russia), 7 μL of ddH_2_O (Syntol, Moscow, Russia), 1 μL of MgCl_2_ and 1 μL (80 ng/μL) of each specific primer. DNA was first denatured for 4 min at 95°C, then 30 cycles of 1 min of denaturation at 94°C, 1 min of hybridization at 62°C, and 1 min of elongation at 72°C, followed by a final elongation step at 72°C for 7 min. PCR products were purified using the Cleanup S-Cap (Evrogen, Moscow, Russia) and the sequencing polymerase reaction was carried out with Big Dye Terminator v 3.1 Cycle Sequencing RR-100 (Applied Biosystems, Vilnius, Lithuania). Polymerase reactions were carried out using 2 μL of purified DNA and 2 μL of primers (12.8 ng/μL). DNA was initially denatured for 1 min at 96°C, followed by 30 cycles of 10 s of denaturation at 96°C, 5 s of hybridization at 50°C, and 4 min of elongation at 60°C. Amplicons were sequenced in both directions using the NANOPHOR-05 capillary DNA sequencer (Syntol, Moscow, Russia). DNA sequences we analyzed using ClustalW 2.0.3 program (53) and BLAST.

### 2.5 Statistical analysis

The mean and standard error of the mean (SE) were calculated using the Student’s t-test for statistical analysis to evaluate the significance of the difference in mortality and pre-rRNA concentration between water-treated Control and experimental groups. All above-mentioned calculations were preformed using Prism 9 software (GraphPad Soft-ware Inc., Boston, USA).

## 3 Results

### 3.1 Mortality of *S. pineti* after contact application of olinscide Schip-11

**Figure 3:**
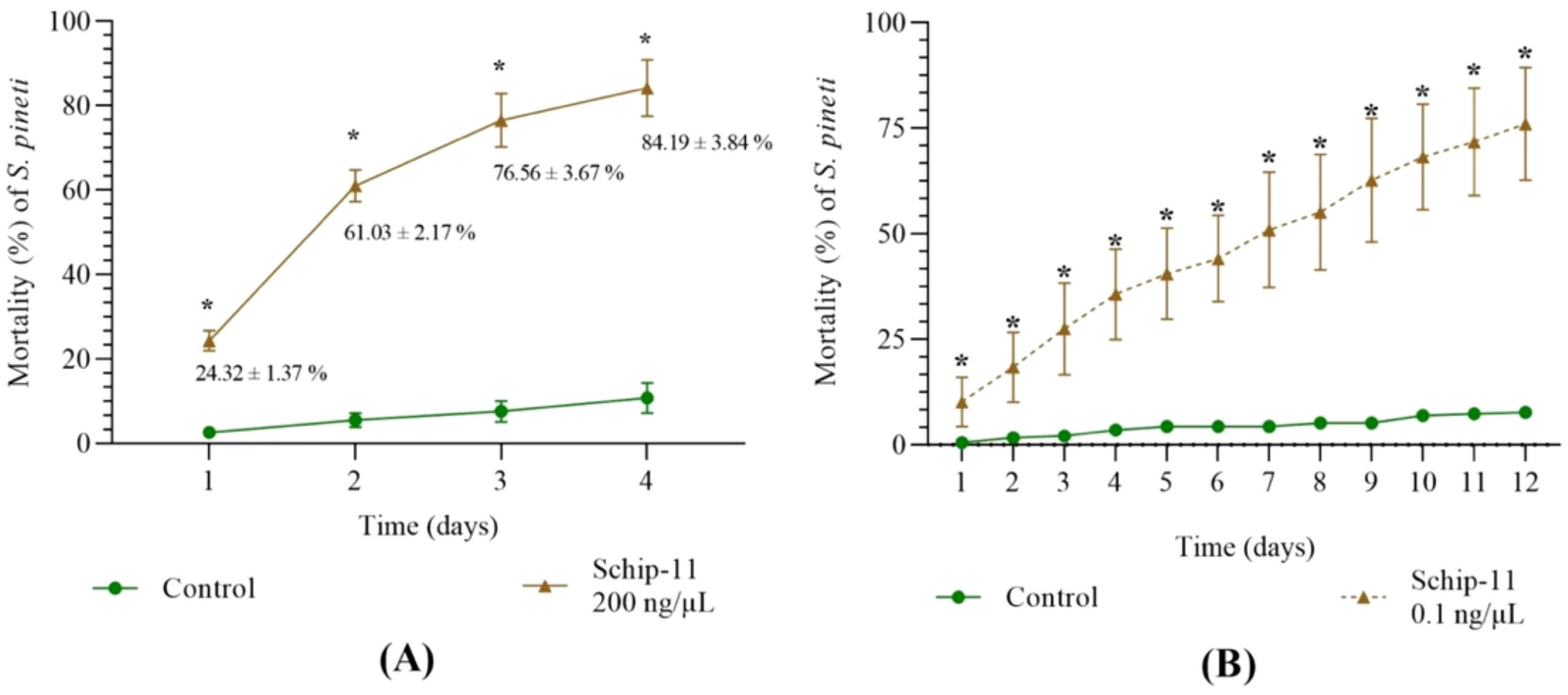
Dynamics of mortality of *S. pineti* after treatment with water and oligonucleotide insecticide Schip-11 in different concentrations: **(A)** 200 ng/µL; **(B)** 0.1 ng/µL; The significance of the difference between Schip-11 group and water-treated Control groups is indicated by *p<0.05.

After treatment of *S. pineti* with olinscide Schip-11 in concentration 200 ng/μL mortality of the pest reached 24.32 ± 1.37%, 61.03 ± 2.17%, 76.56 ± 3.67%, and 84.19 ± 3.84% on the 1^st^, 2^nd^, 3^rd^, and 4^th^ day, respectively (**Figure 3A**) (p<0.05). Of note, the same olinscide (5′-TGT-GTT-CGT-TA-3′; 100 ng/µL) with perfect complementarity to target ITS2 of pre-rRNA also caused significant mortality of closely related species, chrysanthemum aphid *Macrosiphoniella sanborni*. This olinscide caused 67.15 ± 3.32% mortality rate of the chrysanthemum aphid after a single treatment and 97.38 ± 2.49% mortality rate after a double treatment (with daily interval) on the 7^th^ day (p<0.05) (54).

After treatment of *S. pineti* with olinscide Schip-11 in concentration 0.1 ng/μL mortality of the pest reached 10.18 ± 3.36%, 18.44 ± 4.79%, 29.51 ± 5.35%, 35.67 ± 6.19%, and 76.06 ± 7.68% on the 1^st^, 2^nd^, 3^rd^, 4^th^, and 12^th^ day, respectively (p<0.05) (**Figure 3B**). Interestingly, graph of dynamics of mortality of the pest differs from the standard S-curve which is characteristic for olinscides in concentration 200 ng/µL and 100 ng/µL (13, 32, 55, 56) and represents almost linear graph. It should also be noted that insect mortality occurs more slowly when concentration of olinscide is 0.1 ng/µL. Similar insect mortality (≈76%), obtained on 12^th^ day in the group with a concentration of 0.1 ng/μl, was achieved already on the 3^rd^ day in the group with a concentration of 200 ng/μl of olinscide.

Of note, closely related species of aphids from the same subfamily Lachninae, *Cinara pinea* and *Eulachnus rileyi*, also showed high sensitivity to Schip-11 in 0.1 ng/µL concentration. On the 12^th^ day, mortality of *C. pinea* and *E. rileyi* comprised 63.66 ± 19.81% and 67.73 ± 9.16%, respectively in comparison with water-treated Controls (9.14 ± 0.83% and 8.31 ± 2.11%, respectively) (p<0.05). It shows high reproducibility of results and perspective of using low-dose concentrations of olinscides on conifer aphids.

### 3.2 Olinscide Schip-11 significantly decreases concentration of pre-rRNA of S. pineti (investigation carried out on dead insects)

In this article we decided to investigate concentration of pre-rRNA containing target ITS2 region after application of olinscide Schip-11 and used dead individuals of *S. pineti*. We found significantly decreased concentration of pre-rRNA in dead insects treated with water solutions of olinscides in both concentrations, 200 ng/µL and 0.1 ng/µL, compared to dead insects from water-treated Controls (p<0.05).

**Figure 4:**
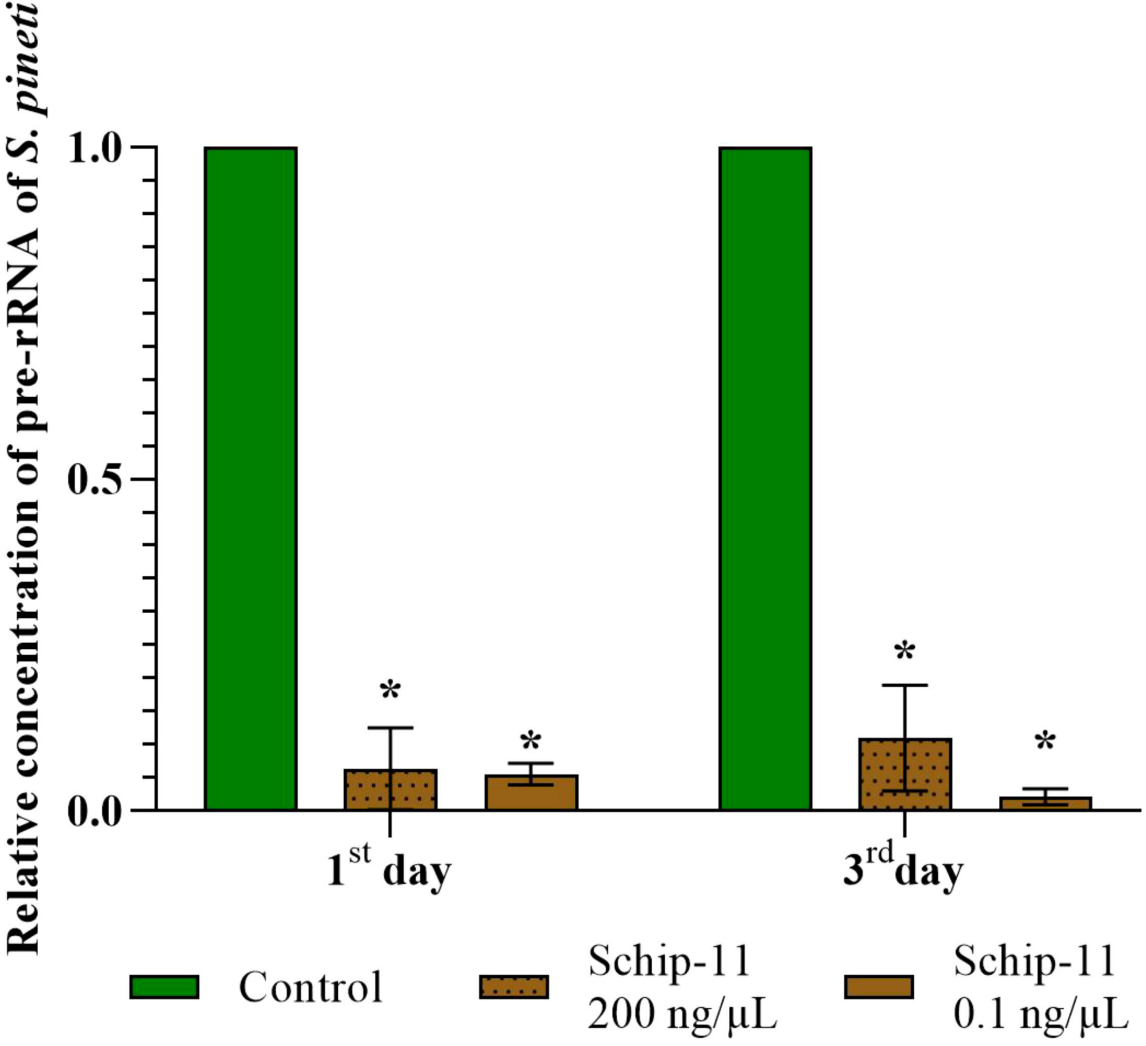
Relative concentration of pre-rRNA of *S. pineti* after treatment with oligonucleotide insecticide Schip-11 in different concentrations (200 ng/µL; 0.1 ng/µL) on the 1^st^ and 3^rd^ day; water-treated Control was taken as 1 (100%). The significance of differences between Schip–11 group and water-treated Controls is indicated by * at p < 0.05.

On the 1^st^ and 3^rd^ day, concentration of pre-rRNA was 15.62 (p<0.05) and 9.09 (p<0.05) times lower compared to water-treated Control for 200 ng/µL of Schip-11. For 0.1 ng/µL group, on the 1^st^ and 3^rd^ day, concentration of pre-rRNA was 17.85 (p<0.05) and 45.45 (p<0.05) times lower compared to water-treated Control (**Figure 4**).

Previously, for survived individuals of chrysanthemum aphid *M. sanborni* we detected hypercompensation of target rRNA and then gradual decrease in 1-3 days after treatment (54) which represents typical reaction of cells of sternorrhynchans on oligonucleotide insecticides through DNA containment mechanism (13, 15). Here we show that dead insect individuals have decreased concentration of target rRNA. It is evident that increased concentration of target rRNA is better than decreased one in survived insects compared to water-treated Control (13), while dead insects are likely to have them decreased (13, 32, 54).

## 4 Discussion

Obtained results show substantial potential of ITS2 regions of pre-rRNAs as a target for olinscides. Moreover, ITS regions of rRNA genes are more variable (**Figure 2**) in comparison with sequences of 5.8S, 18S, and 28S rRNAs (57) allowing the creation of plethora unique sequences of oligonucleotide insecticides. The length of an oligonucleotide insecticide ~ 11 nt makes it possible to create selective oligonucleotide insecticides with a uniqueness frequency equal to 1/4.19·10^6^ and is obviously enough to be used in most agrocenoses (32). In the case of ecosystems with increased diversity, such as forests, it is possible to increase the length of oligonucleotide insecticides to 15–20 nt (9). It is important to note that pre-rRNA and rRNA is a convenient target for olinscides, while mRNA, due to much lower concentration, will be much less susceptible to antisense oligonucleotides, even if oligonucleotide insecticides will possess perfect complementarity to it. Pest rRNA comprises 80% of all RNA in the cell (58) and its use as a target for ‘genetic zipper’ helps making this approach very efficient and selective at the same time. Thousands of different mRNAs make up only 5% of all RNA and use of mature rRNA and pre-rRNA for targeting substantially increases signal-to-noise ratio, ca. 10^5^:1 (rRNA vs. random mRNA) (59).

The use of olinscides could solve, or at least improve, the fundamental problem of insecticide selectivity. The results of our previous work with chrysanthemum aphid *M. sanborni* showed that the change of just one nucleotide at the 1st (5′-end, T to A) and 11th (3′-end, A to T) positions leads to dramatical decrease in biological efficiency of target 11-nucleotides long olinscide Macsan-11 (54). At the same time on scale insects, *Dynaspidiotus britannicus* and *Aonidia lauri*, we showed that non-canonical base-pairing, such as A:С (С:A) and G_olinscide_: U_rRNA_ (52, 60–62) may occur between olinscides and ‘imperfect’ sites of rRNAs of non-target organisms (13). Here for the first time we show that non-canonical base pairing G_olinscide_: U_rRNA_ between target pre-rRNA and olinscide is also well tolerated in aphids. Definitely, non-canonical base-pairing should be taken into consideration during design of olinscides not to harm non-target organisms. Importantly, on olinscides potential hazard for non-target organisms can be calculated, while for conventional chemical insecticides it is impossible task.

Low concentrations of oligonucleotide insecticides (0.1 ng/µl) showed high insecticidal potential against aphids. The detected high mortality rate indicates an effective and target effect of unmodified antisense DNA on the pest. Of note, Yakubov et al. (63) also reported that for low concentrations of oligonucleotides (<0.5 µM), the uptake efficiency by cells is considerably higher and the average concentration of the oligonucleotide derivatives in mammalian cells exceeds the derivative concentration in the medium. This can be understood by assuming that the cells can absorb a limited amount of oligomer on their surface. This could increase the efficiency of the endocytosis process (absorptive endocytosis). At low oligonucleotide concentrations, the contribution of this process predominates. Undegraded oligodeoxynucleotides were found in cellular nuclei and cytoplasm after penetration to cells (63).

It also should be noted that at a comparable concentration (~0.07 ng/μl) dsRNA also have a significant insecticidal effect on the Colorado potato beetle (64). In parallel with oligonucleotide insecticides, a different class of insecticides based on dsRNA is being developed, the action of which is based on RNAi. RNA biocontrols show the best results on coleopterans (65), and much worse on hemipterans (66). In this context, oligonucleotide insecticides and RNA biocontrols as 2 different next-generation classes of insecticides, are able to complement each other’s action in complex preparations for wide range of pests from different orders, especially against those that have shown resistance to many different compounds from major insecticide classes (67).

Obtained here results show perspective of using olinscides against conifer aphids, including *S. pineti*, *E. rileyi*, and *C. pinea*. Using cold fog generators and big cold fogging machines it is possible to treat vast territories of conifer forests without harm to natural enemies (wasps, mites, beetles, etc.) of insect pests and other non-target organisms. Recent results also demonstrated remarkable specificity of oligonucleotide insecticides in action (8, 68) and showed their safety for several non-target organisms: *Quercus robur, Malus domestica* (69), *Triticum aestivum* (70), *Manduca sexta, Agrotis ipsilon* (71), *Galleria mellonella* (8). Aphids form an important part of many food chains and can be part of a healthy garden or forest ecosystem. Thus, olinscides can control a distinct pest species while closely related species will stay unharmed. Using unique complementary sequences to target pre-rRNAs and rRNAs of an insect pest it is possible to create well-tailored olinscides with minimal risks to balance of an ecosystem. As a molecule of natural origin, olinscides do not reduce biodiversity, do not impact soil health, and are not accumulated in ecosystems. We can say that olinscides degrade almost immediately after their action recruiting ubiquitous DNases (8, 13, 54). Studies of the nuclease activity of tissue homogenates of target insect pests (*Lymantria dispar*, *Icerya purchasi*, *Leptinotarsa decemlineata*) and their host plants (*Quercus pubescens*, *Pittosporum tobira*, *Solanum tuberosum*) have shown that most of the used olinscides degrade within 24 hours at 27°C (8, 32, 72) and even faster, within 1 hour, recruiting DNases of *Macrosiphoniella sanborni* (54).

CUAD biotechnology, as well as double-stranded RNA technology, has achieved a significant reduction in the cost of nucleic acid synthesis using innovative methods for production of nucleic acids in vitro (73, 74). СUAD biotechnology has become significantly cheaper due to liquid phase synthesis (72). One of market leaders in liquid phase synthesis, Sumitomo Chemical Co., Ltd. (Tokyo, Japan), offers the synthesis of 1 kg of unmodified oligonucleotides 11 nt long for 25,000 USD (personal communication). In the case of using non-optimized solid-phase DNA synthesis, which is available in many laboratories around the world, including ours, the cost of synthesis of 1 kg of unmodified oligonucleotides (11 nt long) will be ca. 1 million USD. Thus, at a consumption rate of 200 L per hectare, at a concentration of 0.1 mg/L (or 0.1 ng/μL), the price of the required amount of oligonucleotide insecticide will be about 0.5 USD when using liquid-phase DNA synthesis. This price allows to increase the frequency of treatments with oligonucleotide insecticides in real conditions. If non-optimized solid-phase DNA synthesis is used, which is available in many laboratories around the world, including ours, the cost of synthesizing the required amount of oligonucleotide insecticide per hectare will be 20 USD. This price will be very affordable for investigations in the lab.

Of note, today dsRNA-based technology does not have an easy algorithm for creation of pesticides providing selectivity in action and high efficiency like CUAD biotechnology does on several groups of pests (hemipterans and mites) (33). Obviously, long and fragile dsRNAs are more unpredictable in action that is why it is not easy to use them as practical tool for plant protection. In addition to better durability (75) and target specificity, antisense oligonucleotides are easier to synthesize and cheaper than siRNAs or dsRNAs. In the human system, antisense oligonucleotides have been shown to have lower immunoreactivity. For potential applications in edible plants this fact possesses significant importance since accumulation of this kind of means of insect pest control could potentially occur. Importantly, considering potential environmental risks, there are no findings in the numerous human clinical studies that prove, for example, genomic integration events attributable to the use of antisense oligonucleotides (76). While double-stranded RNA insecticides need easy and efficient algorithm and more groups of pests to show their effectiveness on, ‘genetic zipper’ method built on single-stranded DNA insecticides requires only the latter.

Double-stranded RNA biocontrols are perceived as ‘difficult’ insecticides, since they do not have clear and easy algorithm of creation, there is no strategy for RNAi how to avoid target-site resistance in insects, success of their application in the field is unpredictable, affordable production of dsRNA is not publicly available, while publicly available in vitro production is still very expensive (>200 USD/g). The efficiency of a given dsRNA pesticide is largely affected by the selection of the target gene and its targeted region, the size of the dsRNA, the method of dsRNA production and formulation, as well as by the method and dosage of the dsRNA application to crops (77). Undoubtedly, RNAi is an amazing technique for elucidation of gene function but is very fickle tool for insect pest control. On the contrary, CUAD-based oligonucleotide insecticides are considered as ‘easy’ insecticides with simple and efficient algorithm of creation and adaption to potential emergence of target-site resistance (15). Essentially it is about management of minimal risks for the environment and olinscides provide this opportunity, while this idea basically impossible for modern chemical insecticides and long dsRNA, what makes olinscides a unique and perspective approach for plant protection (13). In comparison with dsRNA biocontrols, oligonucleotide pesticides were not only the first contact nucleic acid-based insect pest control agents for plant protection, but also significantly simplified system for creation of efficient and well-tailored insecticides.

The results obtained allow us to look at CUAD biotechnology as a platform capable to occupy a significant part of the insecticide market. Most of the insect pests against which CUAD biotechnology is effective today are representatives from the suborder Sternorrhyncha, which primarily live in the subtropics and tropics, and, to a lesser extent, in the temperate zone (11). Already today, according to our estimations, the ‘genetic zipper’ method is potentially capable of effectively controlling 10-15% of all insect pests using a simple and flexible algorithm of DNA-programmable plant protection. Oligonucleotide insecticides can make a significant contribution to the protection of plants from pests of coffee, cocoa, citrus fruits, cereals, and other important groups of agricultural plants that ensure food security.

## 5 Conclusion

This article for the first time shows that low concentration of oligonucleotide insecticides (0.1 mg/L) leads to increased mortality of aphids. At a consumption rate of 200 L per hectare, the price of the required amount of oligonucleotide insecticide will be about 0.5 USD when using liquid-phase DNA synthesis. In fact, distribution of olinscides in 20 mg/ha ratio gives huge number of molecules per each mm^2^ of hectare, ca. 3.6*10^8^/mm^2^. Thus, each conifer aphid of this size will get enormous amount of contact olinscide molecules. If olinscide molecules have sufficient complementarity (including non-canonical base pairing, like G_olinscide_: U_rRNA_) to target rRNA in pest cells, they will be able to cause death of target insect pest. In the case of using non-optimized solid-phase DNA synthesis, the price of the required amount of oligonucleotide insecticide per hectare will be 20 USD. ‘Genetic zipper’ method based on CUAD biotechnology opens up new frontiers for the large-scale implementation of oligonucleotide insecticides as the next generation class of insecticides in plant protection (8, 13, 14, 40). Oligonucleotide insecticides have a low carbon footprint (72), high safety for non-target organisms, rapid biodegradability in ecosystems, and avoidance of target-site resistance (8, 13, 32, 54, 72). Moreover, we are now able to predict the effectiveness of oligonucleotide insecticides on various insect pests based on their effectiveness in closely related species (15).

The CUAD platform is a simple and flexible DNA-programmable biotechnology for creation of oligonucleotide insecticides (15). Investigation of efficiency of low concentrations of oligonucleotide insecticides together with auxiliary substances (spreaders, adhesives, penetrators, etc.) will help discover the most optimal formulations for control of wide range of pests. How far we are from the point in plant protection when crop will contain only crop, without traces of chemical insecticides (organic xenobiotics)? One thing is clear, we are on the way to it.

## Data Availability

The original contributions presented in the study are included in the article/supplementary material. Further inquiries can be directed to the corresponding author.
